# Differences in Electromyographic Activities and Spatiotemporal Gait Parameters between General and Developed Insoles with a Toe-Grip Bar

**DOI:** 10.1155/2020/6690343

**Published:** 2020-12-18

**Authors:** Teppei Abiko, Shin Murata, Yoshihiro Kai, Hideki Nakano, Dai Matsuo, Michio Kawaguchi

**Affiliations:** ^1^Department of Physical Therapy, Faculty of Health Sciences, Kyoto Tachibana University, Kyoto, Kyoto, Japan; ^2^ASICS Trading Company Limited, Kobe, Hyogo, Japan

## Abstract

The present study was aimed at comparing the muscle activities and gait parameters between the toe-grip bar insoles and general insoles during walking using randomized crossover design. Twelve healthy men participated in this study. Temporal and spatial gait parameters and electromyography (EMG) results were concurrently collected while the subjects walked along an 8 m walkway with the developed and general insoles. Developed insoles provide a three-dimensional mesh structure at the toe portion and a convex bulging structure (toe-grip bar) near the center of the proximal phalanx of the first to fifth toe. The linear mixed model was used to estimate the toe-grip bar insole effect. The results showed that there were no sequence or period effects for any of the examined parameters. During the stance phase, those wearing the developed insoles had significantly higher %IEMG for the TA, GM, and GL than those wearing the general insoles (TA: 5.03%IEMG, *p* = 0.005; GM: 4.65%IEMG, *p* = 0.046; and GL: 6.50%IEMG, *p* = 0.008). During the swing phase, those wearing the developed insoles had significantly higher activity for only the TA compared to those wearing the general insoles (5.54%IEMG, *p* = 0.011). With respect to gait parameters, those wearing the developed insoles had greater step length (2.81 cm, *p* = 0.038), longer stance time (0.03 s, *p* = 0.001), and shorter swing time (−0.02 s, *p* = 0.003) compared to those wearing the general insoles. The results suggest that walking with toe-grip bar insoles contributes to increased crural muscle activity and step length.

## 1. Introduction

The foot, which is among many supporting parts of the body, is the only part that comes in contact with the ground. Moreover, the foot absorbs impact from the ground during walking and running and provides propulsion and balance control during standing, walking, and running by gripping the ground using the toes. This is achieved by toe-grip muscle activities at the late stance phase of the gait cycle [1, 2]. However, the relationship between toe-grip muscle activity and gait parameters remains unclear.

Studies have shown that toe-grip strength decreases with age and is associated with walking and balance ability [3, 4]. Moreover, reports have shown that elderly individuals have significantly lower toe-grip strength compared to healthy adults [3]. Toe-grip weakness has been considered an important risk factor for falls [5, 6] given that elderly individuals who experienced falls had that significantly decreased toe-grip strength compared with those who did not [7]. Our previous study showed that exercise interventions aimed at increasing toe-grip strength, such as towel gathering, decreased the fall rate [8]. However, the regular exercises can be impeded by personal, environmental, and activity characteristics (i.e., towel gathering) [9]. Thus, a variety of interventions to increase toe-grip strength are needed.

We had previously developed and investigated the effectiveness of insoles with a toe-grip bar that made flexing the toes easier [10–15]. Accordingly, healthy young women who wore shoes with toe-grip bar insoles exhibited reduced center of gravity sway [10]. Moreover, a randomized control trial, which compared the effectiveness of general insoles with that of the developed toe-grip bar insoles [11], revealed that the group using the developed insoles displayed better toe-grip strength and flexibility compared to that using the general insoles. Our previous results also showed that toe-grip bar insoles improved toe-grip strength and postural sway among elderly women [12]. Another randomized clinical trial among children that compared the effects of the general insoles with those of the developed insoles [13] showed that running speed increased significantly after 1 month of wearing the shoes with the developed insoles. Thus, it is apparent that the toe-grip bar insoles have the ability to enhance toe-grip strength, as well as posture control and locomotion. However, no study has yet determined whether the toe-grip bar insoles improve toe-grip muscle activity during gait. Furthermore, changes in gait parameters during walking with the developed insoles have remained unknown.

In recent years, geriatrics has shown the importance of sarcopenia, which is the loss of muscle mass and muscle weakness [14]. Newman et al. [15] have established the association between activity level and sarcopenia in men, while it was not shown in women. If walking with a toe-grip bar insole can unconsciously increase muscle activity during walking, it may be one of the strategies to prevent sarcopenia in men.

The present study was aimed at comparing the muscle activities and gait parameters between the developed insoles and general insoles during walking. We hypothesize that the toe-grip bar insole increases the muscle activity of the ankle joint and the step length.

## 2. Materials and Methods

### 2.1. Subjects

A total of 12 healthy men (mean age ± standard deviation (SD): 40.1 ± 8.9 years, mean height: 171.2 ± 2.8 cm, mean bodyweight: 67.5 ± 7.8 kg, and BMI: 23.0 ± 2.5) participated in this study. All participants were recruited from Kyoto Tachibana University. Only men were recruited given the gender differences in toe-grip strength and electromyographic (EMG) activity [3, 16–18]. Participants who reported foot pain within the previous 6 months, had any prior foot surgery, presented with congenital or acquired feet deformities upon clinical examination, or had any other disability that affected their gait were excluded. None of the participants had cardiovascular, neurologic, or musculoskeletal disease. The study was conducted according to the principles of the Declaration of Helsinki and was approved by the local Institutional Ethics Committee (Kyoto Tachibana University). All subjects provided informed written consent for study participation and were free to withdraw from the study at any time.

### 2.2. Procedure

A randomized crossover design was used in which participants were randomly divided into two sequences of insole conditions using random numbers generated by Microsoft Excel 2010 (Microsoft, Redmond, WA, USA). Sequence 1 wore shoes with general insoles first, while group B wore shoes with toe-grip bar insoles first, with both groups swapping shoes after 1 week, which was set as the washout period. All participants were blinded to the group in which they were included.

**Figure 1:**
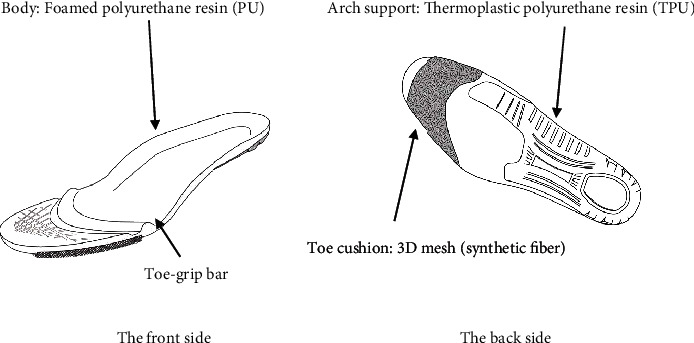
The developed insole with a toe-grip bar.

The developed insoles were molded using synthetic resin foam, a highly repulsive three-dimensional mesh (synthetic fiber) at the toe portion of the back of the insole, and a highly rigid thermoplastic polyurethane resin from the rear end of the first phalanx to the hind foot portion (Figure 1). A three-dimensional mesh structure at the toe portion and a convex bulging structure (toe-grip bar) near the center of the proximal phalanx of the first to fifth toe are provided. The arch support made of thermoplastic polyurethane resin was mounted in order to hold the inside vertical arch in the middle stage of the stance. The general insoles were modeled similarly, except without a toe-grip bar or toe section made from synthetic fiber. Subjects wore the same standard shoes provided by the study investigators.

The three-dimensional mesh structure of the toe portion and the convex bulging structure (toe-grip bar) near the center of the proximal phalanx of the first to fifth toe are illustrated. The arch support made of thermoplastic polyurethane resin was mounted to hold the internal vertical arch during the middle stage of the stance.

Subjects walked along an 8 m walkway with the developed and general insoles. At each walking session, they performed one familiarization trial and two experimental trials, during which temporal and spatial gait parameters and EMG results were concurrently collected.

### 2.3. Measurements and Data Analysis

The OptoGait photoelectric cell system (OptoGait, Microgate, Bolzano, Italy) consisting of light-transmitting and light-receiving bars was used to measure spatiotemporal gait data [19]. Each bar is 1 m in length and is composed of 100 light-emitting diodes that continuously transmit to an oppositely positioned bar. Accordingly, when subjects pass between two bars positioned parallel to the ground, their feet block transmission and reception. Moreover, this system determines the distance and duration time and automatically calculates spatiotemporal parameters. OptoGait was linked via an interface unit to a personal computer using the OptoGait software. Data were extracted at a sampling frequency of 1000 Hz. The present study measured mean walking speed (m/s), cadence (steps/min), step length (cm), stance time (s), and swing time (s) during 2 experimental sessions.

Muscular activity was measured using a surface electromyogram (TeleMyoG2; Noraxon Inc., USA). The electromyogram surface electrodes (blue sensor; Ambu Inc., Denmark) were attached after hair removal and skin abrading over the right tibialis anterior (TA), right gastrocnemius medialis (GM), and right gastrocnemius lateralis (GL) muscle bellies according to SENIAM [20]. Electrodes of the TA were placed at one-third of the line between the tip of the fibula and the tip of the medial malleolus. Electrodes of the GM were placed at the most prominent bulge of the muscle. Electrodes of the GL were placed at one-third of the line between the head of the fibula and the heel. The interelectrode distance was 2 cm from center to center. The reference electrode was then fixed to the head of the right fibula. Signals were sampled at 1000 Hz, after which they were bandpass filtered (20 to 500 Hz) using analysis software (MyoResearch XP; Noraxon Inc., USA) and transferred to a personal computer. Isometric maximum voluntary contractions (MVCs), which were executed twice for all recorded muscles against manual resistance for 3 s using manual muscle testing grade 5, had been performed for normalization. The TA was measured while generating maximal isometric dorsiflexion in the sitting position against plantar flexion resistance. The GM and GL were tested while maintaining a raised right heel standing in the extended-knee position against shoulder pressure. MVC data during the middle 1 s of the entire 3 s measurement were selected, after which mean values of each muscle were calculated.

The obtained EMG signals were subjected to full-wave rectification and then normalized to 100% of the walking cycle time in order to obtain integrated EMG (IEMG) data in the stance and swing phases using OptoGait data, which was synchronized to the EMG system. The obtained IEMG data of the stance and swing phases were normalized to the MVC values of each muscle (%IEMG). Mean values of four strides during 2 experimental sessions were calculated for the representative data.

### 2.4. Statistical Analysis

Results are displayed as mean and standard deviation (SD). The comparison between the groups of participants' characteristics was performed using the Mann-Whitney *U* test. A general linear mixed model was used to estimate the toe-grip bar insole effect (treatment effect). The model contained the fixed effects for the toe-grip bar insole, sequence (order of the insole), and period (order of time). The effect of the sequence could indicate an unequal carryover effect from one insole to the other insole, which would complicate the estimation of the treatment effect. The effect of the period may indicate familiarization to the study procedure, and it was included to obtain an unbiased estimate of the treatment effect. The level of significance was set at *p* < 0.05. All statistical analyses were performed using SPSS version 25.0 (IBM, Chicago, IL, USA).

## 3. Results

There were no significant differences between the groups in any of the descriptive variables (Table 1). The analysis revealed that there were no sequence or period effects for any of the examined parameters. Mean %IEMG values for general and developed insoles are summarized in Table 2. During the stance phase, those wearing the developed insoles had significantly higher %IEMG for the TA, GM, and GL than those wearing the general insoles (TA: 5.03%IEMG, *p* = 0.005; GM: 4.65%IEMG, *p* = 0.046; and GL: 6.50%IEMG, *p* = 0.008). During the swing phase, those wearing the developed insoles had significantly higher activity for only the TA compared to those wearing the general insoles (5.54%IEMG, *p* = 0.011).

Mean gait parameter values for the general and developed insoles are outlined in Table 3. Accordingly, those wearing the developed insoles had greater step length compared to those wearing the general insoles (2.81 cm, *p* = 0.038). Moreover, those wearing the developed insoles had significantly longer stance time (0.03 s, *p* = 0.001) and shorter swing time (-0.02 s, *p* = 0.003) compared to those wearing the general insoles, while no difference in cadence was observed.

## 4. Discussion

The present study sought to determine whether differences in muscle activities and gait parameters existed between the developed toe-grip bar insoles and general insoles. Accordingly, our results showed that walking with the toe-grip bar insoles significantly increased %IEMG activities of the TA, GM, and GL during the stance phase, %IEMG of the TA during the swing phase, and step length. These results supported our hypothesis.

During the late stance phase, the center of the foot pressure shifts toward the forefoot, increasing forward propulsion through flexor pollicis longus and flexor digitorum longus tension and muscle activity [1, 2]. Although previous studies have shown that the function of toe flexor muscles is particularly important for this forward propulsion [5], measuring the activity of such muscles during walking is difficult given their location in the plantar and deep layers of the foot and lower leg. Soma et al. [21, 22] who investigated the muscle activities of the ankle joint during toe gripping while seated showed a significant positive correlation between toe-grip strength and muscle activities of the TA and GM. The study explained that the TA acted as a fixator of the ankle joint during toe gripping, while the GM acted as a cooperative muscle. Therefore, the activity of the crural muscle could be regarded as representative of both ankle joint function and toe gripping during walking. We believe that the increased activities of the crural muscle during the stance phase might have been facilitated by the toe-grip bar and various toe fibers, which allowed for easier toe gripping.

Fast-paced walking requires the generation of more high-peak power and greater functional reserve compared to usual-paced walking [23–25]. Studies have reported that fast-paced walking promoted a greater increase in TA EMG activity during the swing phase than normal-paced walking. These results assume that higher TA activity is required to quickly swing a leg and maintain toe clearance, which is necessary to prepare for faster heel contact. The present study found that those wearing the developed insoles had a shorter swing time than those wearing the general insoles, but there was no change in walking speed. Therefore, the developed insoles might have promoted significantly higher %IEMG activity in only the TA.

The present study found that walking with toe-grip bar insoles increased step length. Moreover, the developed insoles promoted a significantly longer stance time and shorter swing time compared to the general insole. Our insoles were designed such that the toe-grip bar was near the center of the proximal phalanx to facilitate toe-grip movements. In addition, the heel of the insole was slightly elevated, while the toe was made of synthetic fibers (solid mesh) that compressed under the load of the toes during the late stance phase. This configuration supposedly moved the center of the foot pressure forward, resulting in increased step length. Tsujino et al. [26] reported a positive correlation between forward displacement of the center of the foot pressure and the downward vertical force exerted by the toes. Previous studies had reported that forefoot pressure during the terminal stance was associated with push-off movement [27] and that the plantar flexor muscle was a major contributor during this phase [28]. Menz et al. [5] reported that toe flexor strength was associated with plantar forces and pressures during walking among older individuals residing in a retirement village. Moreover, another study showed that although toe-grip strength was not related to cadence, it showed a relationship with walking speed, step length, and stance time [29]. While increasing toe-grip strength did not affect cadence even among young individuals, it did increase walking speed and step length after 6 weeks of training [30].

It has been shown that increased walking speed usually increases muscle activity in the lower leg [23]. In this study, there was no change in walking speed, while muscle activity increased during two conditions. Therefore, the increase in muscle activity might not be affected by the walking speed but by the effect of the developed insole that grasped the toe-grip bar and moved the center of gravity forward.

To the best of our knowledge, no study has examined changes in gait parameters and muscle activities during gait using gripping insoles. Thus, our results support the notion that toe-grip EMG activities have a positive effect on gait and may serve as confirmatory evidence that walking with the developed insoles enhance toe-grip strength and postural sway [10–13]. Since decreased step length in the elderly has been shown to be one of the risk factors [31, 32], walking with the developed insole can be considered an easy and effective method for preventing falls given that it enhances toe-grip strength and step length without special exercise. However, some limitations of the present study need to be noted. EMG activity of the toe flexor muscles during toe griping had not been measured directly. Therefore, this study could not determine the extent to which such muscles had been activated. Second, even with the crossover design, the sample size was small (power: 0.49). Third, the elderly with a risk of fall were not examined. Therefore, further studies should include direct measurements of toe flexor activity and should include more subjects.

## 5. Conclusions

The present study showed that walking with toe-grip bar insoles increased the EMG activities of the TA, GM, and GL during the stance phase but only the TA during the swing phase. Moreover, the developed insoles significantly increased step length. Altogether, the results presented herein suggest that walking with toe-grip bar insoles contributes to increasing the activity of crural muscle as toe-grip muscle and step length independent of the walking speed. It may help prevent sarcopenia and reduce the risk of falls in the elderly.

## Figures and Tables

**Table 1:** Participant characteristics based on the order of intervention.

	Sequence 1 (*n* = 6)	Sequence 2 (*n* = 6)	All participants (*n* = 12)
Age (years)	43.7 ± 10.6	36.5 ± 5.7	40.1 ± 8.9
Height (cm)	170.8 ± 3.1	171.5 ± 2.8	171.2 ± 2.8
Weight (kg)	70.0 ± 5.9	65.0 ± 9.1	67.5 ± 7.8
BMI	24.0 ± 1.9	22.1 ± 2.8	23.0 ± 2.5

Data are presented as mean ± standard deviation.

**Table 2:** Treatment effect of % integrated electromyography values for each muscle during the stance and swing phases between the general and developed toe-grip bar insoles.

Gait phase	Muscle	General insoles	Toe-grip bar insoles	Difference	*p* value
Stance phase	TA	16.01 ± 7.11	21.04 ± 9.38	5.03 ± 4.85	0.005
	GM	24.36 ± 9.28	29.00 ± 14.08	4.65 ± 6.74	0.046
	GL	23.22 ± 12.47	29.72 ± 16.02	6.50 ± 6.47	0.008

Swing phase	TA	21.30 ± 6.96	26.84 ± 9.90	5.54 ± 6.01	0.011
	GM	11.74 ± 6.52	10.05 ± 5.89	–1.69 ± 3.33	0.088
	GL	9.36 ± 5.59	8.89 ± 4.58	–0.48 ± 1.96	0.440

Data are presented as mean ± standard deviation. The general linear mixed model was used. TA: tibialis anterior; GM: gastrocnemius medialis; GL: gastrocnemius lateralis.

**Table 3:** Treatment effect of gait parameters between the general and developed toe-grip bar insoles.

	General insoles	Toe-grip bar insoles	Difference	*p* value
Walking speed (m/s)	1.38 ± 0.11	1.42 ± 0.12	0.05 ± 0.09	0.094
Cadence (steps/min)	110.36 ± 6.14	109.85 ± 6.49	−0.51 ± 3.35	0.592
Step length (cm)	74.79 ± 3.00	77.60 ± 4.42	2.81 ± 3.89	0.038
Stance time (s)	0.63 ± 0.05	0.66 ± 0.05	0.03 ± 0.03	0.001
Swing time (s)	0.46 ± 0.02	0.44 ± 0.03	−0.02 ± 0.02	0.003

Data are presented as mean ± standard deviation. The general linear mixed model was used.

## Data Availability

The data used to support the findings of this study are available from the corresponding author upon request.
